# Scoping review of COVID-19 vaccines access, equity, hesitancy, and uptake in Kenya: lessons for the next pandemic

**DOI:** 10.3389/frhs.2026.1648814

**Published:** 2026-05-13

**Authors:** Vincent Okungu, Stephen Mulupi, Grace Njeri Muriithi, Daniel Malik Achala, Elizabeth Adote, Chinyere Ojiugo Mbachu, Senait Alemayehu Beshaha, Chijioke Osinachi Nwosu, John Ele-Ojo Ataguba

**Affiliations:** 1 Department of Public & Global Health, University of Nairobi, Nairobi, Kenya; 2 Liverpool VCT Care and Treatment, Nairobi, Kenya; 3 African Health Economics and Policy Association, Accra, Ghana; 4 University of Nigeria, Enugu, Enugu, Nigeria; 5 Ethiopian Public Health Institute, Addis Ababa, Ethiopia; 6 University of the Free State, Bloemfontein, South Africa; 7 University of Manitoba Max Rady College of Medicine, Winnipeg, MB, Canada; 8 Partnership for Economic Policy, Nairobi, Kenya; 9 University of Pretoria, Pretoria, South Africa

**Keywords:** COVID-19 vaccine +Access +Kenya, COVID-19 vaccine +Distribution+ Kenya, COVID-19 vaccine +Equity+ Kenya, COVID-19 vaccine +Hesitancy+ Kenya, COVID-19 vaccine +Uptake+ Kenya

## Abstract

**Introduction:**

The global rollout of COVID-19 vaccines was hampered by hesitancy, even as the pandemic intensified, underscoring the urgent need to strengthen vaccination efforts to reach most of the population. The primary objective of this review was to identify the critical barriers to equitable and timely access to and uptake of vaccines in Kenya with a view to mitigating the impacts of future pandemics, such as COVID-19.

**Methods:**

A digital search was conducted between February and March 17, 2024, and between December 2025 and February 2026, using four databases: PUBMED, Google Scholar, Cochrane Library, and Africa Journals Online. The search was limited to peer-reviewed articles only. The keywords used in the search were: COVID-19 vaccine + Access +Kenya; COVID-19 vaccine +Uptake+ Kenya; COVID-19 vaccine +Equity+ Kenya; COVID-19 vaccine +Hesitancy+ Kenya. The search was restricted to studies published from 2020, when the first vaccines were rolled out. Three researchers independently reviewed articles for inclusion and exclusion.

**Results:**

A total of 60 articles were included in the review. The main themes identified include geographic access, socio-demographic factors, economic and political determinants, and distrust in government systems. Among the critical determinants of COVID-19 vaccine uptake, confidence in the vaccine's efficacy and safety was a significant consideration. Geographic access to vaccination sites hindered uptake; for example, only 9.7% were vaccinated in a hard-to-reach county, compared to 53% in an urban county. Sociodemographic factors, including age, gender, level of education, occupation, and co-morbidity, significantly influenced vaccine uptake in Kenya. Above all, the Ministry of Health's failure to mount a convincing response to myths and misconceptions about the COVID-19 vaccine ultimately affected vaccination coverage nationwide.

**Conclusion:**

Future preparedness must ensure inclusive vaccine strategies led by governments with clear policies and tailored outreach. Communication should address vulnerable groups and reduce hesitancy. Improving access and engaging communities are key to equity. Social workers help build trust and local relevance.

## Introduction

Access to vaccines, medicines, and other essential health products is highly inequitable in many parts of the world. The inequities have been exacerbated by socio-demographic and economic factors, which have resulted in poorer health outcomes for women and girls, national, ethnic, religious, racial, and linguistic minorities, indigenous populations, poor people, LGBTQ people, persons with disabilities, migrants and stateless persons, and other groups experiencing marginalization (1). Unfortunately, the COVID-19 pandemic further reinforced the existing inequities in vaccine distribution and access. This prompted the re-emergence of debates on scaling up research and development capacity for vaccines, particularly in Africa, as a proactive measure to enhance domestic vaccine production and counter existing inequities (1).

Based on the stated inequities, this review deepens understanding of the pandemic preparedness ecosystem. The primary objective of this review was to identify the critical barriers to equitable and timely access to and uptake of vaccines in Kenya with a view to mitigating the impacts of future pandemics, such as COVID-19. The assessment of COVID-19 vaccine uptake and hesitancy should provide valuable lessons learned, enabling the successful rollout of new vaccines and helping reduce inequities in vaccine manufacturing and distribution through research, advocacy, and capacity-building activities in Africa.

In perspective, the COVID-19 pandemic had a significant impact on Africa as a whole, with over 9 million infections and more than 200,000 confirmed deaths by the first half of 2022.

Despite the rapid development and deployment of COVID-19 vaccines, most low- and middle-income countries (LMICs) could not achieve at least 10% population coverage in the early stages of vaccine rollouts (2). From the date of the first COVID-19 vaccine product introduction on December 14, 2020, about 5.47 billion vaccine doses have been administered to date, and up to 56% of the global population is vaccinated with a complete primary series of COVID-19 vaccines. Of this percentage, about 28% have received at least one booster dose of the vaccine (2). However, the distribution of these vaccinations was uneven, as epitomized by the low vaccination rates in LMICs, where approximately 15% of the population received at least one dose by the end of 2022 (3). The countries with the highest vaccine uptake rate include Puerto Rico (100%), Nicaragua (97%), the Cayman Islands (95%), Cuba (95%), Portugal (95%), and Peru (93%). The countries with the lowest vaccine uptake against COVID-19 include Haiti (5%), Papua New Guinea (4%), and Yemen (4%) (2). The contribution of vaccines in reducing COVID-19 prevalence has been immense, with approximately 135,000 cases reported worldwide by Q1 of 2024 (2).

Despite the rollout of billions of COVID-19 vaccines in LMICs, inequities in access persisted between lower- and higher-income countries, as well as among different population groups within countries and regions. As of 2022, only 16% of people in LMIC had received at least one dose of the vaccine, compared to 80% in high-income countries. These inequities were linked to the poor global distribution of vaccines, vaccine hesitancy and poor uptake, and a lack of access related to cultural, socio-demographic, and geographic parameters (2).

In Kenya, there were over 337,000 confirmed cases and upwards of 5,600 deaths (4). Vaccine inequity was associated with a lack of transparency, including poor coordination and inconsistent messaging, making it difficult for some population groups to decide whether to take the vaccine. There were also rural-urban differences in access to trusted information channels about the vaccine, as well as myths and misinformation, to which the vaccination program did not have a meaningful response and was poorly equipped to address community concerns (5, 6). Rajshekhar et al. (7) have reported significant variability in vaccine uptake among different age groups in Kenya's urban slums. According to their findings, older individuals are more likely to be vaccinated because they are more risk-averse. A study by Anino et al. among individuals aged 58 to 98 years found that advanced age and chronic disease were associated with increased vaccine hesitancy. At the same time, the distance to vaccination centers was the primary factor contributing to delayed vaccination (8). Access to traditional media (radio, TV, newspapers) was considered an avenue to reduce vaccine hesitancy nationwide (9, 10).

Beyond inequities in vaccine access, the global rollout was also hindered by vaccine hesitancy, even as the COVID-19 pandemic intensified between 2019 and 2022, underscoring the urgent need to strengthen vaccination efforts to reach most of the population. Hence, implementing the “Equitable Access to the COVID-19 Vaccines in Africa” (ECOVA) project should provide valuable lessons to bridge Africa's vaccine access and equity gap and inform preparations for the next pandemic and rollout of new vaccines.

The review focuses on Kenya as part of a larger study involving several other African countries. The focus on Kenya provides the opportunity for in-depth analysis of the factors influencing vaccine equity and uptake in a particular context.

## Materials and methods

### Search strategy and analysis

The study employed a scoping review approach because it can identify trends and gaps in the existing knowledge base to inform research, policy, and practice (11). In essence, a scoping review is ideal for mapping the existing literature on a broad topic such as vaccine hesitancy and uptake. This approach identifies knowledge gaps and determines the feasibility of a focused systematic review to clarify concepts and inform future research agendas (12). A digital search was conducted between February and March 2024 and between December 2025 and February 2026, using four databases: PubMed, Google Scholar, the Cochrane Library, and Africa Journals Online. We snowballed the references of the included articles to assess other studies. A further online search for non-peer-reviewed literature was conducted. Keywords used in the search were as follows: COVID-19 vaccine +Access +Kenya; COVID-19 vaccine +Uptake+ Kenya; COVID-19 vaccine +Distribution+ Kenya; COVID-19 vaccine +Equity+ Kenya; COVID-19 vaccine +Hesitancy+ Kenya. The search was restricted to studies published from 2020, when the first vaccines were rolled out. All studies considered satisfactory in reported findings on COVID-19 vaccine access, equity, hesitancy, and uptake were assessed for inclusion in the review.

In Google Scholar, the following filters were used for each of the word combinations “Relevance”, “Year of publication”, and “Any type” of article. In PubMed, the following filters were used: “Abstract”, “Full text”, “Best match”, and “Year of publication” (See details in Annex 1).

This scoping review followed the analytic framework of Arksey and O'Malley (13). Arksey and O'Malley developed a five-stage methodological framework to guide researchers in conducting scoping reviews (14). The following five-stage framework was used: (1) identifying the research questions, (2) searching for relevant studies, (3) selecting studies, (4) charting the data, and (5) collating, summarizing, and reporting the results (13). The results were organized into themes around vaccine access, equity, hesitancy, and uptake.

### Inclusion and exclusion criteria

All quantitative, qualitative, and mixed-methods academic/published journals (peer-reviewed journals) and unpublished journals between 2019 and 2024 were assessed for relevance. Full-text studies that primarily discussed or evaluated COVID-19 vaccine uptake, equity, hesitancy, or resistance in Kenya were included in the review. The research also included multi-country studies that involved Kenya but excluded studies on COVID-19 vaccine programs in Africa that did not focus on Kenya. No studies were excluded based on language, as all were written in English. The titles and abstracts of the articles deemed relevant to the study were transferred to an Excel sheet to better organize the search results. Duplicates were identified and removed. All reviewers independently read the titles and abstracts to identify the relevant articles to include in the review. Only relevant full-text articles were included in the review.

Critical appraisal and risk of bias assessments are not required in scoping reviews (15). However, as suggested by methodologists such as Levac et al. (12), a quality appraisal was considered; i.e., three team members assessed all literature abstracts for quality and relevance based on the Joanna Briggs Institute Checklist (Annex 2). From relevant studies, the following data were extracted: author name and year of study, country of study (Kenya), study design and setting, participants, age, the outcome of interest, and critical findings.

## Results

**Figure 1:**
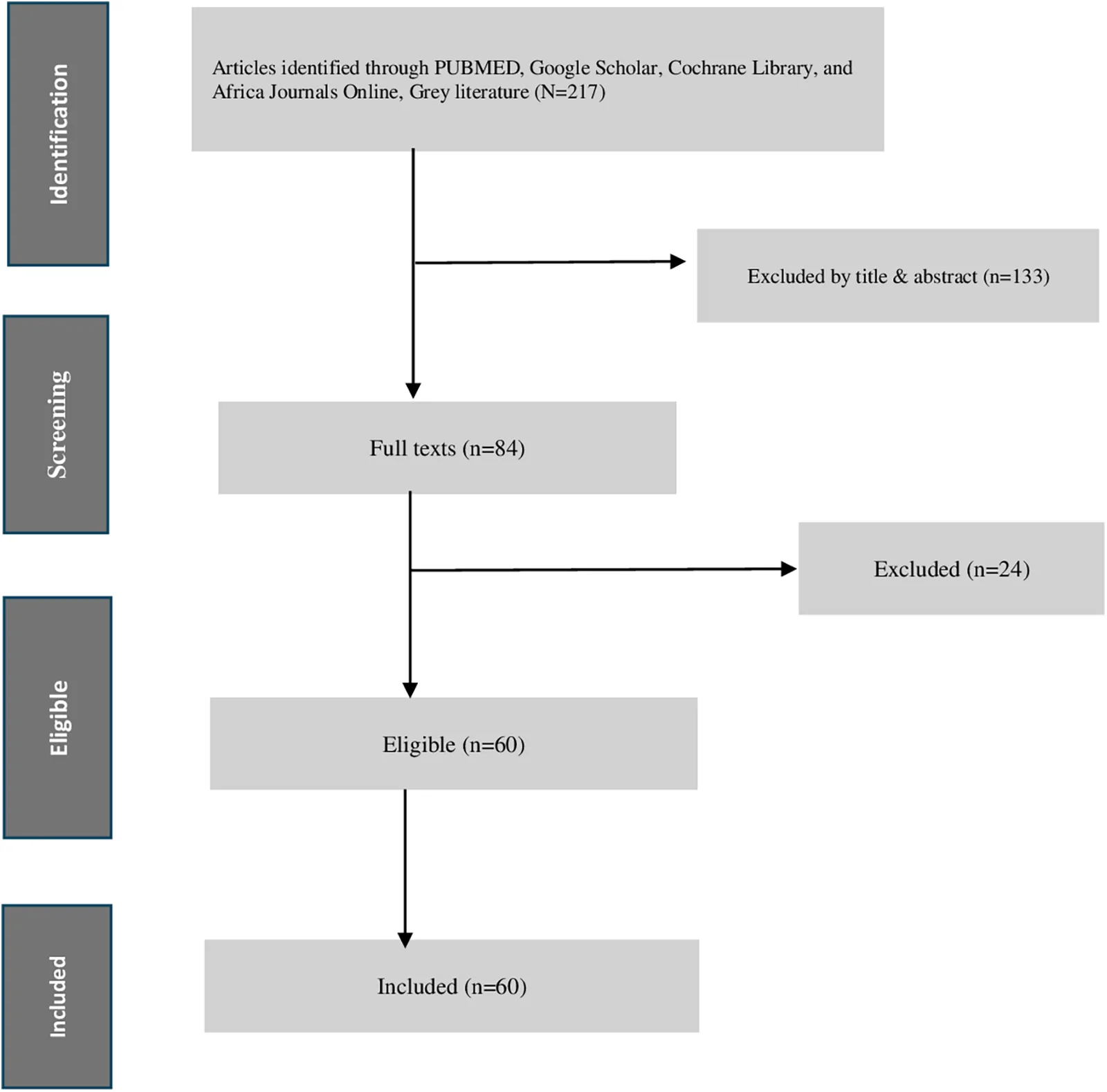
.

Up to 217 articles were set aside for further screening; 60 were included in the review. The review checklist is depicted in the PRISMA diagram (Figure 1).

A summary of the key features of the selected studies is presented in Table 1.

**Table 1:** A summary of the key features of studies included in the scoping review.

Item	Authors & Title	Year	Country	Design	Population
**1.**	Abdulla, S., Mohamed, H., Chweya, R., Wafula, C. & Karanja, S. 2025. Predictors of COVID-19 Vaccine Uptake among Adults in Kiambu, Kenya. *World Journal of Vaccines*, 15, 39–50	2025	Kenya (Kiambu County)	Cross-sectional Survey	Adults
**2.**	Abdulle, H., Masika, M. & Oyugi, J.O. COVID-19: Knowledge, perception of risk, preparedness and vaccine acceptability among healthcare workers in Kenya	2021	Kenya	Cross-sectional	Healthcare workers in Kenya
**3.**	Ackah, B. B. B., Woo, M., Stallwood, L., et al. COVID-19 vaccine hesitancy in Africa: a scoping review. *Global Health Research and Policy,* 7, 21.	2023	Kenya (Mombasa and Kilifi)	Qualitative grounded theory	Healthcare providers, administration personnel, teachers, and individuals with chronic conditions a
**4.**	Akoth, C.O., Ściborski, Romuald J et al. Delay of Acceptance of Covid-19 Vaccine Among Health Care Workers in Western Kenya	2023	Kenya	Cross-sectional survey	Health workers
**5.**	Al-Aghbari, A. A., Naanyu, V., Luchters, S., et al. Reducing Barriers to COVID-19 Vaccination Uptake: Community Ideas from Urban and Rural Kenya	2022	Kenya	Grey literature	All groups
**6.**	Anino, C. O., Wandera, I., Masimba, Z. O. et al. Determinants of Covid-19 vaccine uptake among the elderly aged 58 years and above in Kericho County, Kenya: Institution based cross sectional survey	2023	Kenya (Kericho County)	Institution-Based Cross-Sectional Survey	Elderly people aged 58 years and above
**7.**	Ayieko, S., Jaoko, W., Opiyo, R. O. et al. Knowledge, Attitudes, and Subjective Norms Associated with COVID-19 Vaccination among Pregnant Women in Kenya: An Online Cross-Sectional Pilot Study Using WhatsApp	2024	Kenya	An Online Cross-Sectional Pilot Study Using WhatsApp	Adult population
**8.**	Binyaruka, P., Mtenga, S. M., Mashasi, I. et al. Factors associated with COVID-19 vaccine uptake among people with type 2 diabetes in Kenya and Tanzania: a mixed-methods study	2023	Kenya & Tanzania	Mixed-methods study	Comorbid adults (Type-II diabetes)
**9.**	Blackburn CC, Abdullahi LH, Callaghan T, et al. Examining COVID-19 Vaccine Hesitancy in Nairobi, Kenya, Using the Modified 5 Cs Model	2025	Kenya	Qualitative design using the modified 5 Cs model for vaccine hesitancy	Adult key informants in Nairobi County
**10.**	Carpio, C. E., Sarasty, O., Hudson, D. et al. The demand for a COVID-19 vaccine in Kenya	2021	Kenya	Contingent valuation (CV) of secondary data	N/A
**11.**	ECD/AFDB The Development and Humanitarian Response to the COVID-19 Pandemic in Kenya (2020–2022)	2025	Kenya	Report	Evaluation study
**12.**	Eriksson, J. *COVID-19 vaccine uptake among female students at Pwani University in Kilifi, Kenya.*	2023	Kenya	Cross-sectional survey method	Female university students
**13.**	Essoh, T. A., Adeyanju, G. C., Adamu, A. A. et al. Exploring the factors contributing to low vaccination uptake for nationally recommended routine childhood and adolescent vaccines in Kenya	2023	Kenya	Exploratory Qualitative study	National and county-level vaccine stakeholders
**14.**	Ferrara, G., Mudhune, S., Rogers, A. et al. Willingness to be vaccinated against COVID-19 and associated factors in Migori County, Kenya	2025	Kenya (Migori County)	Secondary data analysis	
**15.**	Grand Synergy Development Initiative (GSDI)	2022	Kenya	Media survey	Muslim and Somali communities
**16.**	Johns Hopkins Center for Communication Programs. COVID behaviors dashboard.	2024	115 countries	COVID-19 Trends and Impact Survey	All
**17.**	Muchangi, J.M., Moraro, R., Omogi, J. et al. Behavioural and Social Predictors of COVID-19 Vaccine Uptake among Persons with Disabilities in Kenya	2024	Kenya	Convergent parallel mixed method study	Persons with disabilities
**18.**	Kilima, J.I., Morema, E.N. and Ochieng, E.O. Uptake of COVID-19 vaccination among healthcare providers in Busia County in Kenya	2023	Kenya	Community-based cross-sectional design	Adults
**19.**	Kimolo, K., Kariuki, J. G. & Karenga, S. M. Determinants of COVID-19 vaccine uptake among adults in Mwala Sub-county, Machakos County, Kenya.	2023	Kenya (Machakos County)	Cross-sectional survey	Adults
**20.**	Kimotho, S. G. 2025. Role of risk perceptions and vaccine hesitancy on decision-making among low-income mothers in Kenya: a qualitative study	2025	Kenya	Qualitative design	Low-income mothers
**21.**	Koech, A., Wanje, O., Mwashigadi, G. et al. “Now that the baby is out, I can be vaccinated”: a qualitative study on COVID-19 vaccine hesitancy in pregnant women in Kilifi, Kenya	2026	Kenya (Kilifi County)	Qualitative design	Pregnant women
**22.**	Limaye, R. J., Paul, A., Gur-Arie, R., Zavala, E. et al. A socio-ecological exploration to identify factors influencing the COVID-19 vaccine decision-making process among pregnant and lactating women	2022	Kenya	Exploratory survey	Pregnant and Lactating Women
**23.**	Limaye, R. J., Sauer, M., Njogu, R. et al. Characterizing Attitudes Toward Maternal RSV Vaccines Among Pregnant and Lactating Persons in Kenya: Key Considerations for Demand Generation Efforts for Vaccine Acceptance	2023	Kenya	Cross-sectional survey	Pregnant and Lactating Persons
**24.**	Matemu, A. & Abong’o, C. COVID-19 Vaccine Equity Project—Kenya Addressing COVID-19 vaccine hesitancy in Kenya. in Kenya.	2022	Kenya	Media article	All groups
**25.**	Ministry of Health. National COVID-19 Vaccines Deployment and Vaccination Plan, 2021	2021	Kenya	Policy paper	Everyone
**26.**	Ministry of Health. Kenya COVID-19 vaccination program- Daily Situation Report: Monday 16th May, 2022. Nairobi: Ministry of Health.	2022	Kenya	Daily situation reports	Everyone
**27.**	Kilima, J. I., Morema, E. N. & Ochieng, E. O. Uptake of COVID-19 vaccination among healthcare providers in Busia County in Kenya	2023	Kenya (Busia County)	Cross-sectional survey	Healthcare providers
**28.**	Mahenzo, L. & Kuhenza. Persons with Disabilities: The Forgotten Vulnerable Group in Kenya's Fight Against Covid-19	2021	Kenya	Blog	Persons with Disabilities
**29.**	Matemu, A. & Abong’o, C. COVID-19 Vaccine Equity Project—Kenya Addressing COVID-19 vaccine hesitancy in Kenya.	2022	Kenya	Media article	All groups
**30.**	Muchiri, S. K., Muthee, R., Kiarie, H. et al. Unmet need for COVID-19 vaccination coverage in Kenya	2022	Kenya	Cross-sectional survey	Adults
**31.**	Mudhune, V., Ondeng'e, K., Otieno, F. et al. Determinants of COVID-19 Vaccine Acceptability among Healthcare Workers in Kenya: A Mixed Methods Analysis	2023	Kenya	Mixed Methods Analysis	Health workers
**32.**	Murage, A., Tan, H. L., Otiso, L. et al. How has Kenya responded to the gendered impacts of COVID-19?	2022	Kenya	Mixed Methods Survey	Adults
**33.**	Naanyu, V., Okwaro, F., Gichere, I. et al. Qualitative exploration of factors associated with COVID-19 vaccination among pregnant women in Kenya	2026	Kenya	Qualitative design	Pregnant women
**34.**	Naidoo, D., Meyer-Weitz, A. & Govender, K. Factors Influencing the Intention and Uptake of COVID-19 Vaccines on the African Continent: A Scoping Review.	2023	Africa	A Scoping Review	N/A
**35.**	Njororai, F., Nyaranga, K. C., Cholo, W. et al. Correlates of Covid-19 Vaccine Acceptance and Hesitancy in Rural Communities in Western Kenya.	2023	Kenya	Cross-sectional survey	Rural communities
**36.**	Ndukui J. Gakunga, Makumi, I. & Affey, F. Hesitancy To Covid-19 Vaccine Uptake in Sub-Saharan Africa: A Systematic Review	2025	Sub-Saharan Africa	Systematic review	N/A
**37.**	Niño, L., Kiragga, A., Miller, F. D. et al. Mapping Structural Barriers: A Geospatial Assessment of Covid-19 Vaccine Inequities In Kenya	2025	Kenya	Secondary data analysis from a demographic survey	N/A
**38.**	Ochieng’, F. A. & O'mathuna, D. 2024. Kenyans’ Perceptions of the Risks of COVID-19 Vaccines: A Scoping Review.	2024	Kenya	Scoping review	N/A
**39.**	Odongo, D. O., Osir, E. & Awandu, S. S. 2024. An evaluation of physical access barriers to COVID-19 vaccines uptake among persons with physical disabilities in Western Kenya.	2024	Kenya	Evaluation study	People with physical disabilities
**40.**	Okello, P., Ogello, V., Thuo, N. et al. COVID-19 vaccine hesitancy among health providers at Kenyatta National Teaching and Referral Hospital Nairobi-Kenya	2024	Kenya	Cross-sectional survey	Health workers
**41.**	Orangi, S., Mbuthia, D., Chondo, E. et al. A process evaluation of the implementation of COVID-19 vaccine deployment in Kenya	2026	Kenya	Evaluation study	N/A
**42.**	Orangi, S., Mbuthia, D., Chondo, E. et al. A qualitative inquiry on drivers of COVID-19 vaccine hesitancy among adults in Kenya.	2024	Kenya	Qualitative design	Adults
**43.**	Orangi, S., Pinchoff, J., Mwanga, D. et al. Assessing the Level and Determinants of COVID-19 Vaccine Confidence in Kenya	2021	Kenya	Cross-sectional Survey	
**44.**	Osur, J., Muinga, E., Carter, J. et al. COVID-19 vaccine hesitancy: Vaccination intention and attitudes of community health volunteers in Kenya	2022	Kenya	Cross-sectional study	Community health volunteers
**45.**	Osur, J. O., Chengo, R., Muinga, E. et al. Determinants of COVID-19 vaccine behaviour intentions among the youth in Kenya: a cross-sectional study	2022	Kenya	Cross-sectional study	Youth
**46.**	Oyekale, A. S. 2021. Compliance Indicators of COVID-19 Prevention and Vaccines Hesitancy in Kenya: A Random-Effects Endogenous Probit Model	2021	Kenya	A Random-Effects Endogenous Probit Model	N/A (secondary data from adults)
**47.**	Oyekale, A. S. 2022. Indicators of Mental Health Disorder, COVID-19 Prevention Compliance, and Vaccination Intentions among Refugees in Kenya. *Medicina (Kaunas),* 58.	2022	Kenya	Cross-sectional study	Refugee populations
**48.**	Rajshekhar N, Pinchoff J, Boyer CB, et al. Exploring COVID-19 vaccine hesitancy and uptake in Nairobi's urban informal settlements: an unsupervised machine learning analysis of a longitudinal prospective cohort study from 2021 to 2022	2023	Kenya	Unsupervised machine learning analysis of a longitudinal prospective cohort study	Urban informal settlement communities
**49.**	Rego, R. T., Kenney, B., Ngugi, A. K. et al. COVID-19 vaccination refusal trends in Kenya over 2021	2023	Kenya	Longitudinal study	Adults
**50.**	Rego, R. T., Reneau, K., Zhukov, Y. et al. Evaluating self-reported vaccination hesitancy in mobile phone surveys in low- and middle-income countries: learned lessons from Ethiopia, Indonesia, Kenya, and Malawi.	2025	Kenya & Malawi	Evaluation study	N/A
**51.**	Schue, J.L., Fesshaye, B., Miller, E. et al. COVID-19 vaccine preferences for pregnant and lactating women in Bangladesh and Kenya: a qualitative study	2024	Bangladesh & Kenya	Qualitative design	Pregnant and lactating women themselves, community gatekeepers or family members, healthcare workers, and policymakers.
**52.**	Schue, J. L., Okwaro, F., Gichere, I. et al. COVID-19 vaccine attitudes and behaviors among pregnant women in Nairobi, Kenya with diverse socio-economic and educational backgrounds	2025	Kenya	Cross-sectional survey	Pregnant women
**53.**	Shah, J. & Rego, R. Understanding and Addressing Vaccine Hesitancy in Kenya: Community Dialogue near Mariakani Hospital in Kaloleni, Kenya.	2022	Kenya (Mombasa County)	Community dialogue	Adult community members
**54.**	Shah, J., Abeid, A., Sharma, K. et al. Perceptions and Knowledge towards COVID-19 Vaccine Hesitancy among a Sub-population of Adults in Kenya: An English Survey at Six Healthcare Facilities	2022	Kenya	Cross-sectional survey	Adults
**55.**	WHO. Immunizing the public against misinformation.	2020	Global	Survey Report	All groups
**56.**	WHO. Kenya Increases Uptake and Equity for COVID-19 Vaccinations.	2021	Kenya	Commentary	All groups
**57.**	Wamalwa, E. M. W. 2023. COVID-19 Vaccination Uptake in Kenya.	2023			
**58.**	Yego, J., Korom, R., Eriksson, E. et al. A Comparison of Strategies to Improve Uptake of COVID-19 Vaccine Among High-Risk Adults in Nairobi, Kenya in 2022.	2023	Kenya	Cross-sectional survey	High-risk adults in Nairobi
**59.**	Zavala et al. Lack of clear national policy guidance on COVID-19 vaccines influences behaviors in pregnant and lactating women in Kenya	2022	Kenya	Qualitative design	People living with disabilities, health workers, and policymakers
**60.**	Zoumpourlis, V., Goulielmaki, M., Rizos, E. et al. The COVID-19 pandemic as a scientific and social challenge in the 21st century.	2020	Kenya	Commentary	N/A

### Context of vaccine hesitancy and equity in Kenya

The COVID-19 vaccination rollout in Kenya, like in many parts of the world, encountered several bottlenecks, including inequities in distribution, geographical access, acceptance, and uptake. In its strategy to improve access and equity to COVID-19 vaccines, Kenya's Ministry of Health (MOH) developed and implemented the National COVID-19 Vaccine Development Plan (2021), which prioritized vaccine distribution based on vulnerability, vaccine availability, and health system capacity (16). The implementation of the plan involved partnerships with county governments, funding organizations, and civil society organizations to expand vaccination coverage countrywide. In some areas of the country, such as sections of Nairobi County, the MOH vaccination program offered doorstep delivery of COVID-19 vaccines through outreach initiatives to boost access and uptake. Despite these concerted efforts, the MOH indicated that only 26% of the total population was fully vaccinated as of 2022.

**Figure 2:**
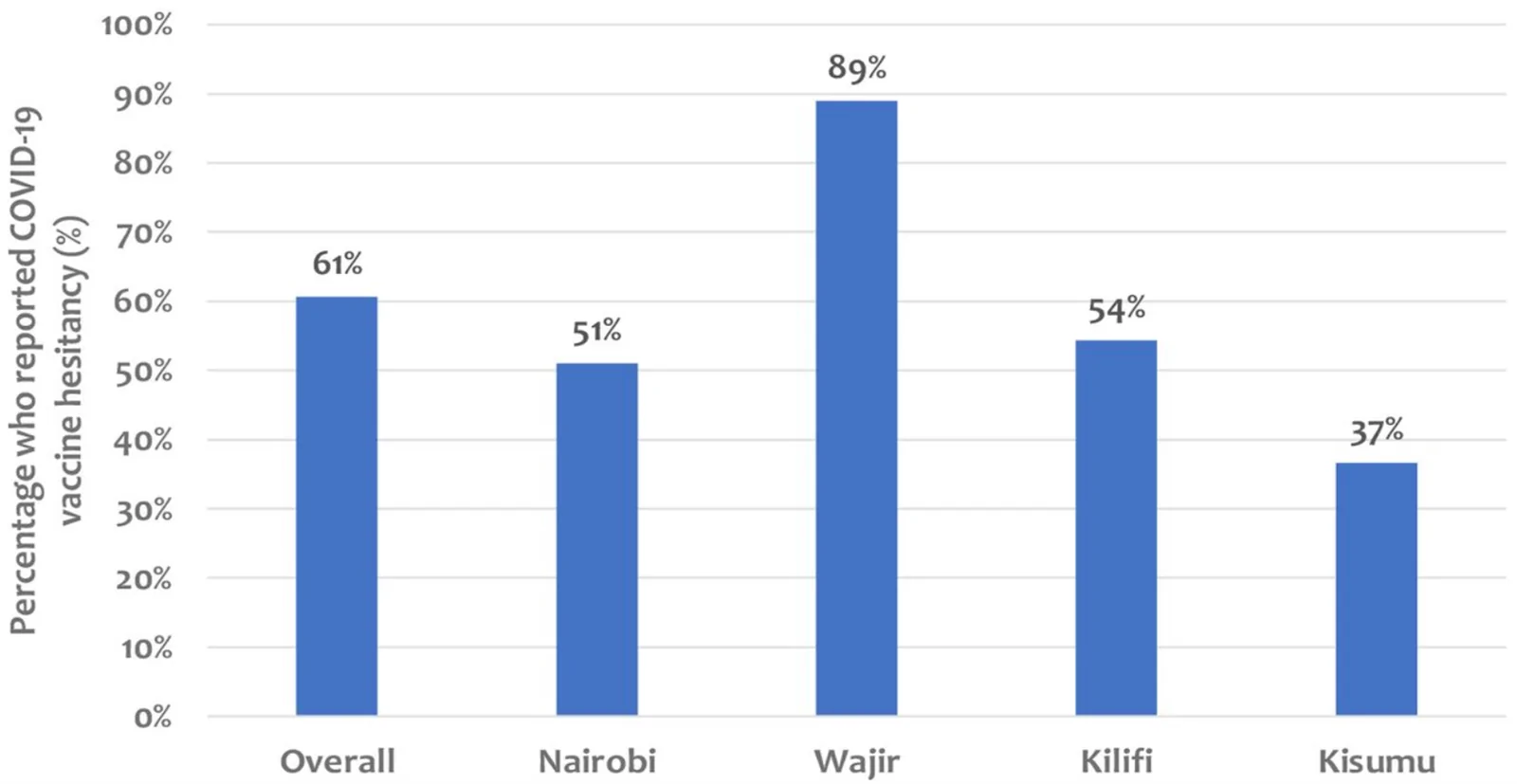
Levels of vaccination hesitancy across study counties. Source: Orangi et al. 2021.

In a study on the levels and determinants of COVID-19 vaccine hesitancy in Kenya, Orangi et al. (9) report a 61% hesitancy rate among adults. Figure 2 shows levels of vaccine hesitancy across study counties.

The findings, as shown in the graph, suggest that marginalized counties such as Wajir, with a high concentration of Muslim adherents, have high hesitancy.

### Thematic issues driving vaccine access, equity, and uptake in Kenya

Box 1 presents the themes identified in the review. The WHO describes vaccine hesitancy as the reluctance or refusal to vaccinate, despite the availability of vaccination services (17). A systematic review by Ndukui J. Gakunga et al. (18) across Africa found hesitancy as a widespread problem. The thematic issues (Table 1) driving vaccine access and hesitancy, with implications for equitable distribution and uptake, were identified from the review and discussed below.

Box 1 **Themes identified in the review.**

• Geographic location and place of residence (marginalized, semi-arid regions, rural, urban residence)
• Sociodemographic factors (e.g., level of education, employment status, age, and marital status)
• Special populations (e.g., health workers and pregnant women)
• People living with disabilities
• Gender
• Age and comorbidity
• Economic and political factors
• Distrust in government
• Myths and religion
• Lack of policy direction
• Individual intentions to vaccinate
• State of mental health

### Geographic access and place of residence as drivers of equity

**Figure 3:**
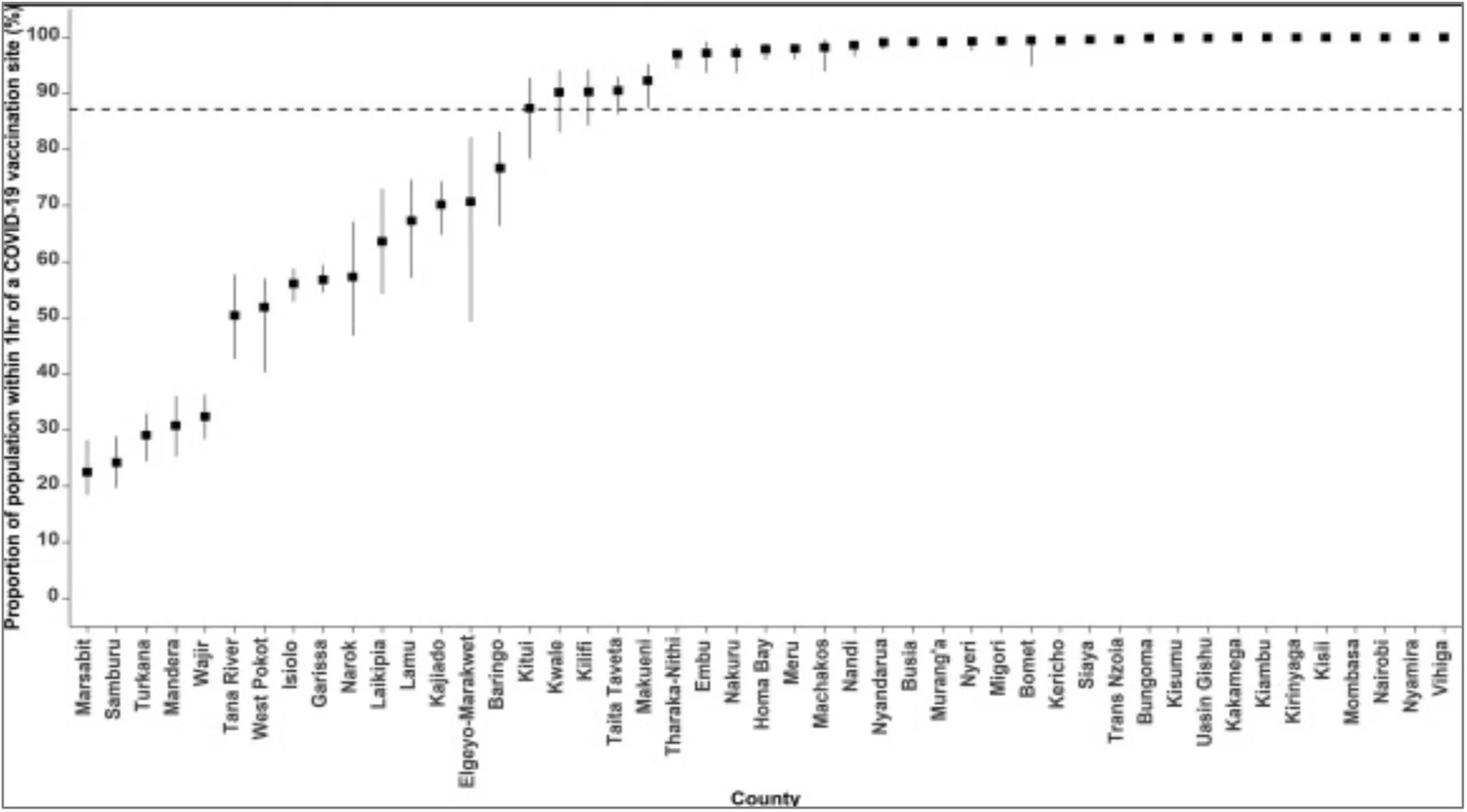
The proportion of the population living within one hour's travel time to a COVID-19 vaccination site. Source: Muchiri et al. 2022.

The COVID-19 vaccines offered through government platforms were free. However, studies have shown that the location and number of vaccination centers imposed financial and physical barriers to the population, particularly for those living in arid and semi-arid areas, in other hard-to-reach places, and for those with physical challenges or residing in densely populated areas. Odongo et al. (19), Orangi et al. (20), and Muchiri et al. Niño et al. (21) found major geographic disparities in vaccination rates ranging from 5.9% in remote counties such as Garissa to 46% in urban clusters such as Nyeri County. Odongo et al. (19), Orangi et al. (20), and Muchiri et al. (22) have acknowledged both direct transportation costs and indirect costs associated with travel and waiting time as key determinants of vaccine access and equity. Muchiri et al. estimated that the time required to reach the nearest vaccination center varied significantly across the country. On average, the travel time was 75.5 min countrywide. According to county estimates, the shortest average travel time was in Nairobi County, at just 5.9 min on average, while the longest was in Marsabit County, at 294.0 min (ranging from 244.9 to 367.7 min) (Figure 3).

About 23% of the 47 counties had an average travel time exceeding two hours. The counties with the longest travel times, ranging from 137.3 to 294.0 min, were also the most marginalized in terms of physical access to vaccination centers (22). There is no evidence of a nationwide vaccine stock-out. The counties with the longest travel time also demonstrated lower vaccine coverage compared to other areas, e.g., Marsabit County (9.7%), Mandera County (10.3%), and Wajir County (11%), compared to Nyeri County (53%), Nairobi (48,5%), and Kakamega (39.2%) (23).

In other findings, Muchiri et al. (22) note that the likelihood of vaccination increased by approximately 28% for individuals residing in urban areas compared to those in rural areas. However, there were marked inequities within urban areas between populations living in formal and informal settlements. Urban areas had the advantage of shorter travel times to vaccination sites, greater vaccine availability, higher literacy rates, and lower vaccine hesitancy—all factors that contributed to higher vaccine uptake (4, 7, 22).

Logistical challenges, including inadequate or uneven distribution of vaccination centers between high- and less-populated areas, and inadequate staff to deliver quality services and administer the COVID-19 vaccine, reduced vaccine uptake, particularly among marginalized and vulnerable groups (19, 24–26). Muchiri et al. (22) observed that the distribution of COVID-19 vaccination centers was uneven, with a concentration in densely populated areas, including Central, Western, Lake Victoria Basin, and Coastal regions, which collectively accounted for 17 counties, each averaging 13 sites, and provided over 50% of the national vaccination capacity. Marginalized arid regions hosted 53 sites, serving 13.7% of the population. In other findings, restrictions on travel and movement were associated with increased vaccine uptake (27). A lack of diverse communication materials and methods in the implementation of vaccination programs also reduced uptake (24).

### Socio-demographic factors influencing equity and hesitancy

Gender, age, disability, level of education, employment status, pregnancy or lactation, and comorbidity were among the key socio-demographic factors influencing vaccine uptake.

#### Employment

Employment in the private sector was associated with a 42% lower vaccine uptake than employment in the public sector (28). However, Osur et al. (29). However, Osur et al. (29) found that employment status, particularly among those working in the private sector and at facilities that offered vaccinations, increased vaccination rates. The contrasting findings suggest a complex web of socio-economic factors influencing vaccine hesitancy and acceptance in different country contexts, which require further research. Blackburn et al. (30) identified vaccination requirements for school and employment as factors that drove vaccination.

#### Education

Binyaruka et al. (31) observed that individuals with tertiary education were four times more likely to be vaccinated than those with no education (AOR = 4.25), and those with health insurance were almost twice as likely to be vaccinated as those without health insurance (AOR = 1.70). Njororai et al. (32) confirm these findings, indicating that vaccine acceptance was lower among those with no formal education (OR: 2.25; *p* < 0.02). Similar findings are reported by Kimolo et al. (33) who report significant correlations between level of education and vaccine uptake. observed that individuals with tertiary education were four times more likely to be vaccinated than those with no education (AOR = 4.25), and those with health insurance were almost twice as likely to be vaccinated as those without health insurance (AOR = 1.70). Njororai et al. (32) confirm these findings, indicating that vaccine acceptance was lower among those with no formal education (OR: 2.25; *p* < 0.02). Similar findings are reported by Kimolo et al. (33) and Schue et al. (34), who observed that the level of education is an important determinant of vaccine uptake even among pregnant women.

#### Special population groups

Several authors, including Mudhune et al. (35), Okello et al. (36), Kilima et al. (37), Ayieko et al. (38), Akoth et al. (39) and Osur et al. (29), have reported very low hesitancy (between 1.2% and 19%) among health workers and CHWs. This is partly due to health workers' knowledge about COVID-19 vaccines that significantly and positively influenced vaccine uptake (OR: 16.3), as well as receipt of information from colleagues (OR: 5) and print media (OR: 4.6) (37). For example, 97.1% of healthcare workers were knowledgeable about COVID-19 vaccines, and those who received information from their colleagues and print media were five times more likely to be vaccinated than those who did not (37).

Special population groups also included pregnant women and lactating mothers. Ayieko et al. (38) and Ayieko et al. (40) show that the odds of vaccination were three times higher among pregnant women aged 30 years or older, and that COVID-19 vaccination among pregnant women was more closely associated with their attitudes towards the vaccine. Similarly, Koech et al. (41) and Naanyu et al. (42), through separate qualitative studies, identified pregnancy as a key driver of vaccine hesitancy. Living with somebody who had symptoms of COVID-19, and having symptoms of COVID-19, as well as vulnerabilities such as refugee status and geography, contributed to vaccine hesitancy (5).

#### Marital status

The positive effect of marital status on vaccine uptake is also reported in other studies (19, 37, 43). Odongo et al. confirm that marriage increases the odds of becoming vaccinated (AOR = 2.2) (19). The positive effect of marital status on vaccine uptake is also reported in other studies (19, 37, 43). Odongo et al. confirm that marriage increases the odds of becoming vaccinated (AOR = 2.2) (19).

#### People living with disability (PLWD)

Individuals with disabilities have been identified as being at greater risk of COVID-19 infection due to difficulties in maintaining basic hygiene practices and social distancing (25). Despite the increased risk of infection, individuals with difficulty in movement and speaking within this population had a significantly lower uptake of COVID-19 vaccines (*p* = 0.001) (19). The directive for social distancing complicated access to vaccines for people with disabilities because of the need for support and institutionalization (26, 44).

#### Gender

**Figure 4:**
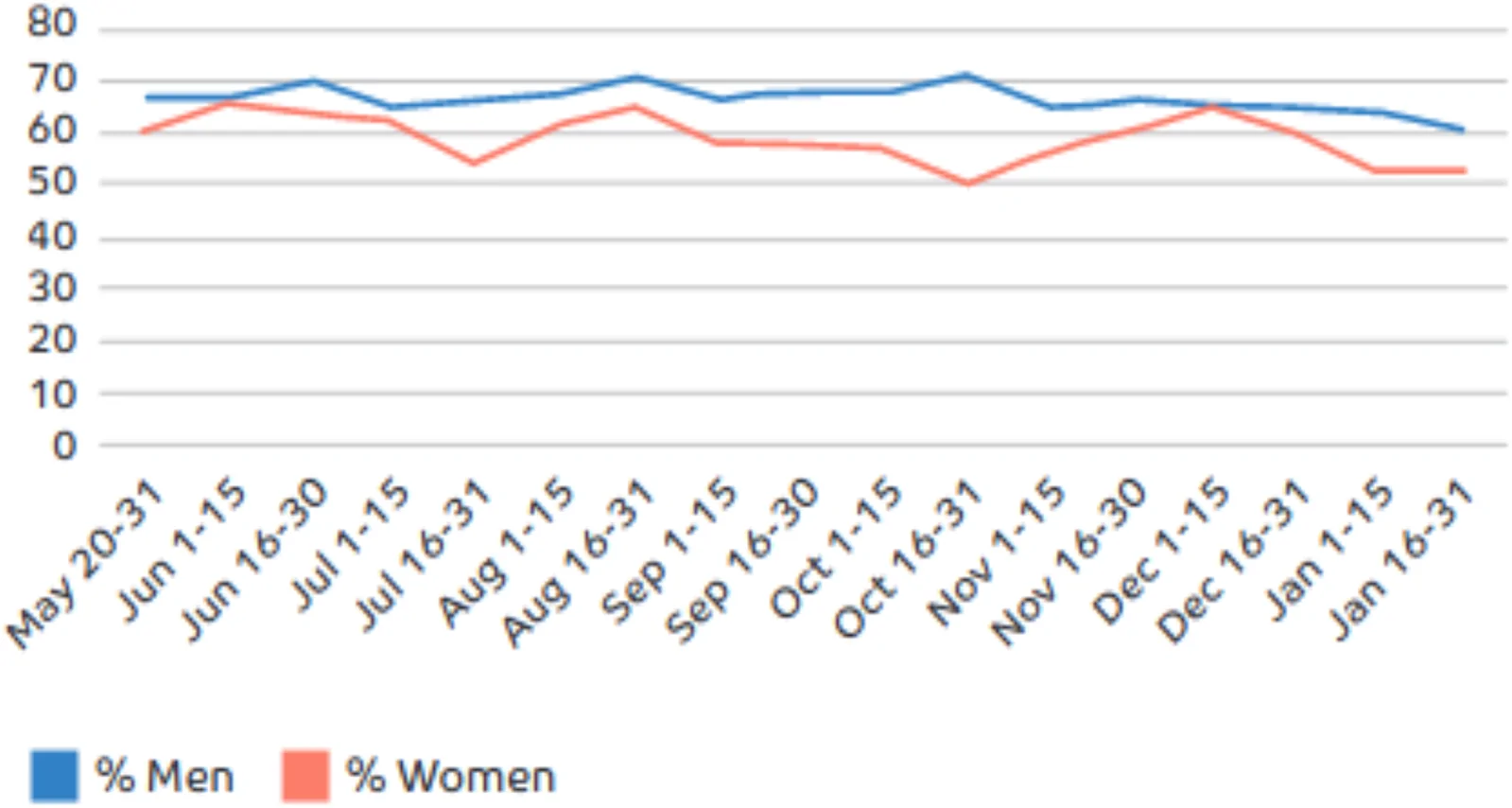
The percentage of men and women respondents who were exposed to COVID-19 information from government health authorities (20 May 2021-31 June 2022 (Kenya). Source: Johns Hopkins center for communication programes COVID-19 behaviour dash board.

There were noticeable gender differences in access to the COVID-19 vaccines in Kenya. In a survey conducted in western Kenya involving 857 participants, Njororai et al. (32) found that vaccine uptake was positively associated with being male (AOR: 1.46, *p* < 0.03). Similarly, reports by the Johns Hopkins Center for Communication Programs COVID-19 Behavior Dashboard indicates that men had greater access to COVID-19 information than women (45), which may have contributed to higher vaccine uptake among men (Figure 4).

On the other hand, more men than women reported higher rates of COVID-19 infections and deaths (46), suggesting greater vulnerability to COVID-19, which should contribute to higher vaccine uptake. Murage et al. (46) noted that while vaccine acceptance was relatively high among both men and women in Kenya, unvaccinated women were less likely to get vaccinated due to structural barriers such as difficulties traveling to vaccination sites, inability to obtain one's preferred vaccine type, lack of vaccination appointment, and difficulties in leaving work and/or school. Similar findings by Kimolo et al. (33) indicate a significant association between sex and vaccine uptake, with more men than women likely to be vaccinated. Women were also relatively alienated from decision-making in response to the COVID-19 pandemic in Kenya and globally. For instance, women were under-represented in the global COVID-19 task forces. In Kenya, only four women were included in the 22-member committee. It is argued that the limited inclusion of women's insights and specialized knowledge from the task forces may have contributed to a less effective pandemic response and influenced how the COVID-19 story and governmental actions were perceived (46).

Other studies that applied a gender lens in the COVID-19 vaccine access and uptake highlighted the connection between the age of women, marital status, and gender-based violence (GBV). Women of middle age, married or divorced, and who reported a higher risk of GBV were more likely than young adults to express a willingness to be vaccinated (50.5% vs. 41.5%), but were possibly not willing to get the vaccine (7). This suggests potential barriers to vaccination for this population group, which social support mechanisms should target to increase coverage.

#### Age and comorbidity

Patients aged 60 years and older or who had other health conditions had higher vaccine uptake than younger individuals (47). However, some studies have highlighted the high prevalence of vaccine hesitancy among elderly populations in Kenya (8). Odongo et al. demonstrated that age (*p* = 0.001) significantly influenced COVID-19 vaccine access and uptake (2024). Compared to older adults, Osur et al., in a survey among the youth (15–35 years), show that the youth were hesitant to vaccinate, with only 42% ready to be vaccinated against COVID-19, 52% waited to see the outcomes of those vaccinated, and 6% refused to receive the vaccine (43). The authors identified several key factors influencing the youth's intentions to get vaccinated, including a lack of comprehensive information about the vaccine, which leads to doubts about its effectiveness and concerns about its health impact (43). Other factors include risk aversion among youth, distrust of the government's intentions regarding the vaccine, and the implications of religious beliefs. These findings show that the main drivers of vaccine uptake among Kenyan youth are modifiable risk factors that can be resolved through targeted, evidence-based engagement strategies (43, 48). On the other hand, adults over 60 or with chronic conditions showed a high vaccination rate of 89%, largely due to their perceptions of the risks of severe illness and death from COVID-19 (31).

Binyaruka et al., in a mixed-methods study of adults with comorbidities, suggest that age, among other factors such as educational background and health insurance coverage, is a key driver of Kenya's higher COVID-19 vaccine uptake among people with type 2 diabetes (T2D) compared with Tanzania (31). However, in a separate study of comorbid patients in a county in western Kenya, the COVID-19 vaccine uptake rate among people with comorbidities was significantly lower than that among those without (*p* = 0.03, OR: 0.3) (37).

### Economic and political drivers of COVID-19 vaccine equity and uptake

The economic dimension of non-free COVID-19 vaccines was explored by Carpio et al., who explained that the larger majority of Kenyans (at least 96%) were willing to accept a COVID-19 vaccine, and about 80% were willing to pay between USD 50 and USD 68 for the vaccines. The authors further state that factors such as the risk of hospitalization and household income significantly affected the willingness to pay for vaccination (49). On the other hand, influential leaders (political and religious) can swing vaccination efforts either way. Binyaruka et al. noted that the explicit political acceptability of the COVID-19 vaccine facilitated a threefold higher vaccine uptake in Kenya compared to Tanzania, where such support was limited (31). In a qualitative study, Orangi et al. observed that influential leaders promoted vaccine uptake and, in some cases, drove hesitancy; for example, when political and religious leaders recommended the vaccine, uptake was higher than when such support was absent (27). Niño et al. (21) and OECD/AfDB (50) have also determined socioeconomic factors as drivers of vaccine uptake. On the other hand, influential leaders (political and religious) can swing vaccination efforts either way.

### Distrust as a key driver of hesitancy

Distrust in COVID-19 vaccines and government intentions was a key driver of hesitancy. In Kenya, while vaccine refusal decreased from 24% to 9% between February 2021 and October 2021, vaccine hesitancy was found to be largely driven by distrust in the government's response to COVID-19 and misinformation surrounding vaccine safety and side effects (5, 7, 28, 30, 35, 46, 51–53). Mistrust in government experts, the science behind the vaccine, and the healthcare system was closely linked to broader concerns about the safety and efficacy of COVID-19 vaccines (3, 4, 24, 30, 54). In addition, Schue et al. (55), Kimotho (56) and Kilima et al. (37) identified drivers of mistrust, including the number of vaccine doses required, negative media coverage, and misinformation about side effects. Abdulla et al. (57) observed a 76% reduction in vaccine uptake among those who believed the COVID-19 vaccine would not protect from infection.

### Myths and religion as drivers of hesitancy

In a survey of CHWs by Limaye et al. (58) the role of myths, interpersonal norms, religion, and eligibility for vaccination were found to be important considerations in the uptake of COVID-19 vaccines among pregnant and lactating mothers. Myths about COVID-19 vaccines have been documented as a leading cause of hesitancy and low vaccination rates in Africa (59). The myths were influenced by socioeconomic status, literacy levels, and religious beliefs (60). Myths about COVID-19 vaccines have been documented as a leading cause of hesitancy and low vaccination rates in Kenya (61) and Africa (59). The myths were influenced by socioeconomic status, literacy levels, and religious beliefs (60).

Among the younger population, the vaccines were intended to introduce microchips into individuals to track recipients. The other myth was that the vaccines would alter the genetic makeup to develop superhumans. The most common myths about the COVID-19 vaccines included the following: that the COVID-19 vaccines contained substances meant to control African populations by causing infertility, as well as spreading HIV and cancer to increase mortality, as a way of controlling the population growth rate (60). Ferrara et al. (62) found that nearly half of the respondents (42.3%) interviewed in Kenya believed that the COVID-19 pandemic was a global conspiracy and refused vaccination. Other authors, such as Kimotho (56), observed that mothers who expressed hesitancy believed the vaccines might cause infertility or long-term health problems.

Among the religious communities, the COVID-19 vaccines were linked to the apocalyptic events where vaccination is likened to receiving the mark of the beast and turning away from the faith, with subsequent eternal damnation (59).

### Lack of policy direction influences hesitancy

The lack of policy direction regarding vaccination of special groups such as pregnant and lactating mothers, comorbid patients, and people living with disabilities (PLWD) was found to have played a role in vaccine hesitancy and uptake. The poor policy interpretation regarding the eligibility of special groups, such as lactating and pregnant mothers, to receive COVID-19 vaccines was highlighted as a barrier to uptake (58, 63). A qualitative study by Zavala et al. (63) reported pervasive uncertainty and a lack of policy direction regarding COVID-19 vaccines for the stated special groups.

Policy and programmatic interventions to address hesitancy are numerous. These include increased education and awareness about COVID-19, engagement of diverse stakeholders, use of diverse materials and methods in the communication and implementation of vaccination programs, increased vaccination facilities and staff capacity to deliver quality services, and revision of relevant government health policies and guidelines (4). The influence of medical staff in promoting immunization is a key factor in reducing vaccine hesitancy is noted (64).

### Individual intentions to vaccinate influenced hesitancy

There is a direct link between health-related intentions and the respective health-related behaviors (65). According to Osur et al. (29) COVID-19 vaccination coverage is low when intentions to vaccinate are low, i.e., when individuals intend or plan to get vaccinated against COVID-19, they are more likely to follow through; the reverse is also true. However, a separate study by Rego et al. (5), indicates that vaccination did not translate into actual vaccination. Some either flatly refused the vaccine or took time to be convinced of the need to vaccinate. Other studies, such as Orangi et al. (20) and Schue et al. (55), identified vaccine preferences as a determinant of individual intentions to vaccinate.

## Mental health Status

Oyekale conducted two separate studies to explore the influence of mental health on COVID-19 vaccine uptake. In their findings, the experience of mental health disorders in the form of nervousness, loneliness, and hopelessness significantly influenced vaccine hesitancy (*p* < 0.10) (66, 67).

### Evidence gaps

There is contrasting evidence that requires further research. For example, while Yego et al. (47) report that patients aged 60 years and older had higher vaccine uptake than younger individuals, other studies, such as Anino et al. (8), have highlighted the high prevalence of vaccine hesitancy among older adults in Kenya. Second, a study by Yego et al. (47) indicates that comorbidities improved the COVID-19 vaccine, but Kilima et al. noted that the COVID-19 vaccine uptake rate of people with comorbidities was significantly lower than that of those without (*p* = 0.03, OR:0.3) (37).

The strong recommendation that efforts to increase vaccine coverage should target social, political, and cultural norms needs to be tested. This needs to consider the local and national context for effective vaccination drives and reaching underserved populations. Lastly, there is currently a scarcity of literature on the impact of mental health on vaccine equity and hesitancy. More qualitative and quantitative research is needed to better understand the link between mental health and vaccine uptake and hesitancy.

## Discussion and recommendations

Achieving high vaccine coverage, particularly with newly introduced vaccines targeting adults, requires a multi-pronged approach involving all key stakeholders, including governments, politicians, healthcare workers, civil society, religious leaders, and local communities. Since barriers to high vaccine coverage manifest at both macro- and micro-levels, a multifaceted strategy, population targeting, and innovation appear to be the most effective approach to reach priority groups with tailored vaccine messages. The WHO identifies advocacy, risk communication, and social mobilization as key strategies to improve vaccine uptake, equity, and reduce hesitancy (68).

Countries differ in context, which drives vaccine uptake and equity. As such, developing strategies to reduce vaccine inequity should consider individual country contexts and cultures (28). Likewise, there were noticeable internal inequities in access to the vaccines in Kenya, with marginalized geographical areas, women, the youth, and people living with disabilities disproportionately affected in accessing COVID-19 vaccines. The gender differences in access to COVID-19 vaccines have been variously documented in different contexts outside Kenya. For example, a systematic review estimated that about 70% of men are likely to get vaccinated against COVID-19, compared to 54% of women (69). Gender is therefore an important driver of vaccine equity across contexts. In a commentary, Nassiri-Ansari et al. (82) argue that gender differences require women- and girls-led groups to be included in the development and design of vaccination programs. Toshkov (70), in a review of 27 European countries, concludes that women are significantly more likely than men to express hesitancy toward COVID-19 vaccination.

Similarly, the distance to vaccination sites and the number of vaccination centers imposed significant barriers to vaccine access. Remote regions and rural areas had fewer vaccination centers than urban areas. The argument was that, because urban areas had a higher population density in smaller spaces, it was necessary to accelerate vaccination drives to control COVID-19 infections. The concentration of vaccination centers in areas with high population density across the country indicated a strategic initiative to rapidly increase vaccine access and uptake. Whilst this kind of targeting is acceptable when services are brought to the population, it can potentially alienate less populated regions and contribute to inequity in the delivery and uptake of COVID-19 vaccines.

To mitigate problems of distance, it is recommended that vaccine delivery strategies should involve outreaches, door-to-door delivery, and increasing the number of vaccination centers (19, 22, 71). These strategies can also improve access to and uptake of COVID-19 vaccines among populations such as PLWD and pregnant women.

Notably, the vaccine uptake followed specific demographic patterns. Age, level of education, comorbidity, pregnancy or lactation status, employment as a healthcare worker or frontline worker, religion, and belief systems were found to influence vaccination acceptance and hesitancy in Kenya. Several studies worldwide (72–77) have confirmed the role of various demographic factors in vaccine uptake. Colvin et al. observed that ethnic minorities were more likely to express vaccine hesitancy than white people (72). Knowledge about the vaccine and its role in reducing COVID-19 severity was found to improve COVID-19 vaccine uptake in a state in the US (72).

Improving vaccine uptake among diverse demographics, including women, youth, individuals with comorbidities, and those with low educational backgrounds, requires targeted interventions with communication strategies that utilize clear, simple, and context-specific language to address specific rumors and misinformation about COVID-19 vaccines (24, 27, 32, 46). Webb and Sheeran (65) recommend that, to strengthen the intention to vaccinate and ultimately improve vaccine uptake, attitudes towards vaccination should be improved, incorporating contextual social norms to support vaccination interventions and ensure vaccine availability. To enhance uptake in marginalized areas, Muchiri et al. (22) suggest interventions such as increasing the number of vaccination centers and conducting vaccination outreaches to institutions.

Some socio-demographic factors, such as comorbidity and working in the private sector, yielded contrasting findings regarding COVID-19 uptake. The contrasting findings can be attributed to differences in geographic settings, sample populations, and the effectiveness of vaccine messaging. In addition, differences in educational achievement could also help explain why more educated regions have higher vaccine uptake, regardless of other demographic characteristics.

Vaccines were accessible to 61% of the Kenyan population, but less than 30% received vaccinations, indicating a persistent issue of vaccine hesitancy. A mix of interventions involving communication that improves public trust is crucial in promoting vaccine acceptance (27, 28, 78). However, vaccine hesitancy is not unique to COVID-19 vaccines, as it has been reported with other vaccines. Everist (79) underscored the impact of hesitancy on the uptake of polio vaccines in South Asia. Concerns about vaccine safety can be linked to vaccine hesitancy, but safety concerns are only one of many drivers of hesitancy. Negative attitudes towards the government, misinformation on vaccine efficacy and safety, mistrust in healthcare professionals or the healthcare system, the role of influential leaders, vaccine access costs, and distance to vaccination centers all contribute to hesitancy (17, 18, 20, 34, 36, 42, 54, 80).

Hesitancy is not limited to specific demographics or geographies. The WHO states that hesitancy was expressed among rural ethnic minorities, remote communities, and wealthy urban residents. Subgroups with religious or philosophical inclinations objected to the vaccine, and, in some instances, higher levels of education either blocked or facilitated vaccine uptake (17). Overcoming hesitancy requires targeted educational campaigns that reach the majority of the target population with evidence-informed COVID-19 vaccination messages. These campaigns should effectively highlight the risks associated with the disease and emphasize the benefits of vaccination, along with other preventive measures (20, 22). The targeted communication is particularly beneficial for the less educated, uninsured, and the youth, who are more vulnerable to misinformation and rumors about the vaccine.

These interventions to address hesitancy, equity, and vaccine uptake should consider the mental health status of the targeted populations. At the same time, there needs to be clarity of policy on the vaccination of special groups such as pregnant and lactating mothers, PLWD, and comorbid populations. Failure to articulate a vaccination policy regarding these groups may increase their risk of severe illness from COVID-19 infection. The policy gap regarding COVID-19 vaccination for pregnant and lactating women was also reported in Bangladesh by Limaye et al. (81), who recommended effective dissemination and understanding of policies.

## Conclusion

Achieving high vaccine coverage during future pandemics requires a coordinated, multi-stakeholder approach led by governments and supported by healthcare workers, civil society, religious leaders, and communities. Central to this effort is the timely dissemination of accurate, tailored information by Ministries of Health to counter misinformation and build public trust. Communication strategies must emphasize the personal and social benefits of vaccination, address specific concerns of vulnerable groups such as women, older people, and those with comorbidities, and use innovative outreach methods to reach priority populations. Advocacy, risk communication, and social mobilization are essential to reduce hesitancy and promote equity. To ensure inclusive vaccine access, preparedness plans must recognize gender dynamics, cultural contexts, and internal inequities. This includes designing delivery systems that are convenient for women, engaging men to support women's health, and prioritizing marginalized groups such as youth, people with disabilities, and remote communities. Clear policy guidance is needed for special populations like pregnant and lactating women. Vaccine availability must be improved by expanding access points, offering flexible hours, and implementing mobile outreach. Social workers and culturally knowledgeable community members should be engaged to support communication, household engagement, and feedback collection, ensuring that strategies are responsive and locally grounded. Ultimately, achieving equitable distribution and high uptake rates is essential to the success of the vaccination campaign worldwide, underscoring the need for continued efforts to address the identified barriers.

## Data Availability

Publicly available datasets were analyzed in this study. This data can be found here: N/A.

## Annex 1 Detailed search strategy and results

**Factors influencing vaccine uptake in Kenya: a scoping review**

**Database search dates:** February and March 2024; December 2025 and February 2026

**Table:**

Word Combination	Hits			
	PubMed	Google scholar	Cochrane library	African journals online
Covid-19 vaccine+ hesitancy+ Kenya	51	9,650	1	36
Covid-19 vaccine+ access+ Kenya	48	17,100	0	207
Covid-19 vaccine+ equity+ Kenya	10	16,600	0	69
Covid-19 vaccine+ uptake+ Kenya	48	16,300	2	125

## Annex 2 JBI Critical Appraisal Checklist for Systematic Reviews and Research Syntheses

Reviewer ______________________________________ Date _______________________________

Author___________________________________Year_________Record Number_______

**Table:**

		Yes	No	Unclear	Not applicable
1.	Is the review question clearly and explicitly stated?	□	□	□	□
2.	Were the inclusion criteria appropriate for the review question?	□	□	□	□
3.	Was the search strategy appropriate?	□	□	□	□
4.	Were the sources and resources used to search for studies adequate?	□	□	□	□
5.	Were the criteria for appraising studies appropriate?	□	□	□	□
6.	Was critical appraisal conducted by two or more reviewers independently?	□	□	□	□
7.	Were there methods to minimize errors in data extraction?	□	□	□	□
8.	Were the methods used to combine studies appropriate?	□	□	□	□
9.	Was the likelihood of publication bias assessed?	□	□	□	□
10.	Were recommendations for policy and/or practice supported by the reported data?	□	□	□	□
11.	Were the specific directives for new research appropriate?	□	□	□	□

Overall appraisal: Include □ Exclude □ Seek further info □
