# Correction: Scoping review of COVID-19 vaccines access, equity, hesitancy, and uptake in Kenya: lessons for the next pandemic

**DOI:** 10.3389/frhs.2026.1974455

**Published:** 2026-09-04

**Authors:** Vincent Okungu, Stephen Mulupi, Grace Njeri Muriithi, Daniel Malik Achala, Elizabeth Adote, Chinyere Ojiugo Mbachu, Senait Alemayehu Beshaha, Chijioke Osinachi Nwosu, John Ele-Ojo Ataguba

**Affiliations:** 1 Department of Public & Global Health, University of Nairobi, Nairobi, Kenya; 2 Liverpool VCT Care and Treatment, Nairobi, Kenya; 3 African Health Economics and Policy Association, Accra, Ghana; 4 University of Nigeria, Enugu, Nigeria; 5 Ethiopian Public Health Institute, Addis Ababa, Ethiopia; 6 University of the Free State, Bloemfontein, South Africa; 7 University of Manitoba Max Rady College of Medicine, Winnipeg, MB, Canada; 8 Partnership for Economic Policy, Nairobi, Kenya; 9 University of Pretoria, Pretoria, South Africa

**Keywords:** COVID-19 vaccine +Access +Kenya, COVID-19 vaccine +Distribution+ Kenya, COVID-19 vaccine +Equity+ Kenya, COVID-19 vaccine +Hesitancy+ Kenya, COVID-19 vaccine +Uptake+ Kenya

The funding of the article processing charges was erroneously omitted. The following text has now been added to the Funding statement:

“The article processing charges for this article were funded by the International Development Research Centre (IDRC). The funder had no involvement in the handling, peer review, or editorial decision for this article.”

The original version of this article has been updated.

